# Elucidation of the interactome of the sucrose transporter StSUT4: sucrose transport is connected to ethylene and calcium signalling

**DOI:** 10.1093/jxb/erac378

**Published:** 2022-09-16

**Authors:** Varsha Garg, Jana Reins, Aleksandra Hackel, Christina Kühn

**Affiliations:** Humboldt-Universität zu Berlin, Institute of Biology, Department of Plant Physiology, Philippstr. 13 Building 12, 10115 Berlin, Germany; Humboldt-Universität zu Berlin, Institute of Biology, Department of Plant Physiology, Philippstr. 13 Building 12, 10115 Berlin, Germany; Humboldt-Universität zu Berlin, Institute of Biology, Department of Plant Physiology, Philippstr. 13 Building 12, 10115 Berlin, Germany; Humboldt-Universität zu Berlin, Institute of Biology, Department of Plant Physiology, Philippstr. 13 Building 12, 10115 Berlin, Germany; Cardiff University, UK

**Keywords:** Calcium binding, calcium channel, calcium inhibition, dual targeting, ethylene perception, protein-protein interaction, sub-cellular dynamics, sucrose transport

## Abstract

Sucrose transporters of the SUT4 clade show dual targeting to both the plasma membrane as well as to the vacuole. Previous investigations revealed a role for the potato sucrose transporter StSUT4 in flowering, tuberization, shade avoidance response, and ethylene production. Down-regulation of *StSUT4* expression leads to early flowering, tuberization under long days, far-red light insensitivity, and reduced diurnal ethylene production. Sucrose export from leaves was increased and a phase-shift of soluble sugar accumulation in source leaves was observed, arguing for StSUT4 to be involved in the entrainment of the circadian clock. Here, we show that StSUT4, whose transcripts are highly unstable and tightly controlled at the post-transcriptional level, connects components of the ethylene and calcium signalling pathway. Elucidation of the StSUT4 interactome using the split ubiquitin system helped to prove direct physical interaction between the sucrose transporter and the ethylene receptor ETR2, as well as with the calcium binding potato calmodulin-1 (PCM1) protein, and a calcium-load activated calcium channel. The impact of calcium ions on transport activity and dual targeting of the transporter was investigated in detail. For this purpose, a reliable esculin-based transport assay was established for SUT4-like transporters. Site-directed mutagenesis helped to identify a diacidic motif within the seventh transmembrane spanning domain that is essential for sucrose transport activity and targeting, but not required for calcium-dependent inhibition. A link between sucrose, calcium and ethylene signalling has been previously postulated with respect to pollen tube growth, shade avoidance response, or entrainment of the circadian clock. Here, we provide experimental evidence for the direct interconnection of these signalling pathways at the molecular level by direct physical interaction of the main players.

## Teaser

An esculin-based transport assay and a systematic screen for protein-protein interactions of StSUT4 revealed that sucrose, ethylene, and calcium signalling are closely linked.

## Graphical Abstract

**Graphical Abstract:**
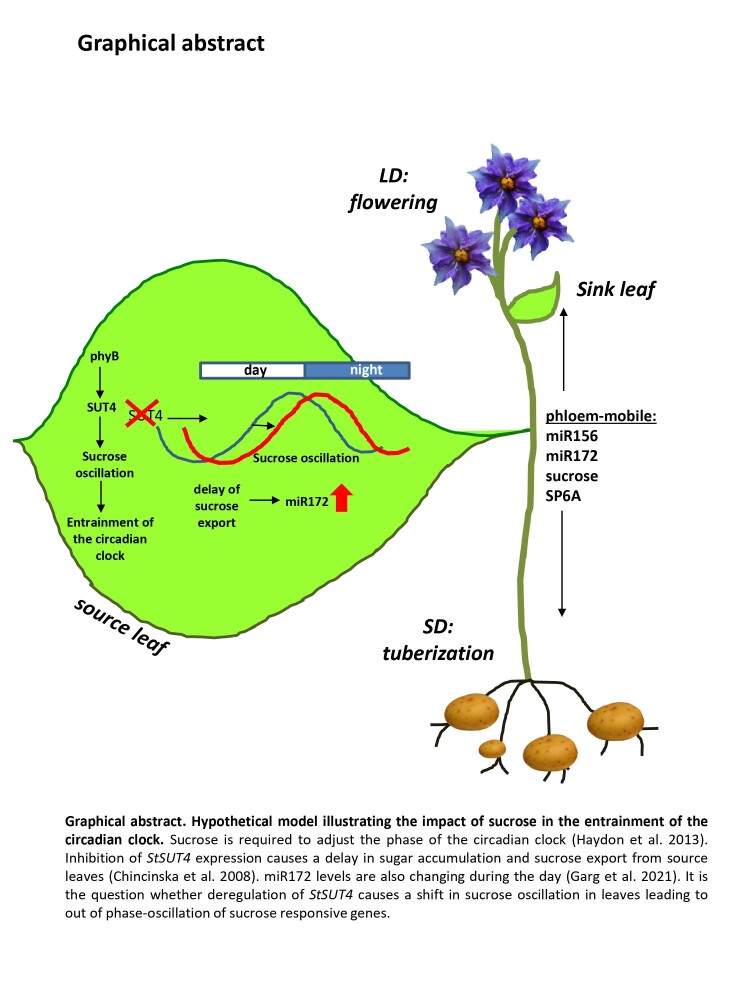

## Introduction

The sucrose transporter StSUT4 from potato is a well-characterized transporter protein whose main function is the inhibition of flower and tuber induction in a sucrose-dependent manner (Chincinska *et al.*, 2008). It has also been shown that StSUT4 is also involved in far-red light perception and the shade avoidance response (Chincinska *et al.*, 2008). It is regulated by the circadian clock, and *StSUT4*-RNAi plants show increased levels of the phloem-mobile *miRNA172* (Garg *et al.*, 2021), and display exactly the same phenotype regarding internode elongation and tuber induction, as described for transgenic potato plants overexpressing *miR172* under control of the constitutive CaMV35S promoter (Martin *et al.*, 2009). *StSUT4* transcript abundance is generally very low (Weise *et al.*, 2000), and transcript stability is regulated at the post-transcriptional level (He *et al.*, 2008; Liesche *et al.*, 2011).

Interestingly, *StSUT4*-silenced potato plants also show reduced ethylene production, which in wild-type potato plants takes place at a very low level, and follows a diurnal rhythm (Chincinska *et al.*, 2013).

In *StSUT4*-silenced potato plants, not only is the diurnal oscillation of ethylene production disturbed, but also the accumulation of soluble sugars in source leaves is somehow delayed, with maximum levels shifted to the dark period of the day (Chincinska *et al.*, 2008). Also, the maximal sucrose export from the leaves into the phloem is delayed and shifted towards the end of the day under long days, with higher amplitude (Chincinska *et al.*, 2008).

Here, we investigate the regulation of *StSUT4* at the post-transcriptional level by mRNA quantification, and at the post-translational level by screening for protein-protein interaction partners, analysis of mutated constructs generated by site directed mutagenesis, and by sub-cellular localization.

Interestingly, we identified a functional member of the family of ethylene receptor proteins, StETR2, which, according to co-expression databases, is tightly co-regulated with StSUT4 (Supplementary Fig. S1), to physically interact with StSUT4 in the split ubiquitin system. Furthermore, the potato calmodulin-1 protein StPCM1 (Poovaiah *et al.*, 1996) was shown to be one of the StSUT4-interacting proteins. Both interactions could be confirmed by alternative methods such as bimolecular fluorescence complementation (BiFC). A third interacting protein has homologies to calcium-load activated calcium channels. The impact of calcium ions on activity of the sucrose transporter StSUT4 was therefore investigated in detail. For these purposes, it was indispensable to establish a reliable transport activity assay for this low affinity transporter that transports sucrose only at very low rate.

An esculin-based transport assay was successfully established for the type III sucrose transporter of the SUT4 clade, that helped to determine transport kinetics in detail. Our investigations suggest a completely different regulatory mechanism and pH dependence for the StSUT4 transporter, compared with other well described type I sucrose transporters such as StSUT1, which was (also) shown to be efficiently inhibited by divalent cations such as calcium or magnesium (Krügel *et al.*, 2013). Interestingly, rising calcium concentrations not only affect StSUT4 activity, but also its sub-cellular localization.

## Materials and methods

### Plant material

Potato (*Solanum tuberosum* subsp. *tuberosum* var. Desiree) and tobacco (*Nicotiana benthamiana*) plants were grown in the greenhouse with a cycle of 16 h light (22 °C) and 8 h darkness (15 °C). Additional illumination was provided by high-pressure sodium lamps SON-T Green Power and metal halide lamps MASTER LPI-T Plus (Philips Belgium, Brussels). Both lamps were distributed equally in the greenhouse. The mean photosynthetic photon flux density was about 150 µmol m^-2^ s^-1^. Transgenic plants of StSU4 (RNAi and overexpressors) used in this study were previously generated in our laboratory (Chincinska *et al.*, 2008).

### Bacterial and yeast strains

*Saccharomyces cerevisiae* L40ccU A [Mat a, His3Δ200, trp1-901, leu2-3,112, lys2: (lexAop)4–HIS3, ura3::(lexAop)8–lacZ, ADE::(lexAop)8–URA, GAL4, gal80, can1, cyh2; Goehler *et al.*, 2004] was used for the split ubiquitin system. Bacterial strains *Escherichia coli* DB3.1 (F^-^, gyrA462, endA1, Δ(sr1–recA) mcrB, mrr, hsdS20(rB^–^mB^-^), supE44, ara14, galK2, lacY1, proA2, rpsL20 (Sm^r^), xyl5, Δleu, mtl1) and *Escherichia coli* NovaXG Zappers (F^–^, mcrA, Δ(mcrC–mrr), endA1, recA1, φ80lacZΔM15, ΔlacX74, araD139, Δ(ara–leu)7697, galU, galK, rpsL, nupG, λ– tonA (Novagen, Darmstadt)) were used for GATEWAY cloning.

Transient transformation of *Nicotiana benthamiana* leaves was performed by infiltration with *Agrobacterium tumefaciens* pGV2260 or *Agrobacterium tumefaciens* EHA105 (Hellens *et al.*, 2000).

### Cloning of the bait construct

The StSUT4 bait construct was amplified via a two-step PCR reaction and cloned into pENTRY and the Cub vectors using the GATEWAY technology (Invitrogen, USA). Primers used for the generation of the bait construct are listed in Supplementary Table S1.

### Construction of a cDNA library from *Solanum tuberosum*

A potato cDNA library was generated from all above and below-ground plant tissues and cloned into the pENTRY1A vector as described (Krügel *et al.*, 2012). The complexity of the cDNA library was 6.3 × 10^5^ and the average insert size was 1.3 kb. All sequenced clones were in the sense orientation. The cDNA library was shuttled into pNXgate32 3HA with inserted *ccdB* gene. Transformation of highly competent *E. coli* (NovaXG Zappers, Novagen, Germany) occurred by electroporation. Cells were plated on 20 Petri dishes (20 cm diameter) and plasmid isolation was performed with Plasmid Mega Kit (Qiagen, Germany).

### GATEWAY compatible SUS vectors

pNXgate and pXNgate are multiple-copy plasmids containing TRP1 for selection in yeast, suitable for NubG-X and X-NubG fusions, respectively, of prey polypeptides X. pMetYCgate is a low-copy plasmid with the selection marker LEU2, comprising the Met-repressible MET25 promoter, B1-KanMX-B2 cassette, and CubPLV. pMetYCgate is suited for Y-CubPLV fusions of bait peptides Y. The MET25 promoter can be used to modulate bait levels.

Bait constructs were cloned into the low-copy vector CubPLV Met25YCgate (Obrdlik *et al.*, 2004). In order to facilitate efficient cloning via *in vivo* recombination, the *ccdB* gene was amplified from pDONR221 (Invitrogen, Karlsruhe, Germany) and ligated via *Pst*I and a blunted *Hind*III site within the GATEWAY cassette of CubPLV met25 pXCgate. Insertion of the 665 bp *ccdB* gene allows selection in *E. coli* DH5α after successful recombination with the StSUT4 bait in pDONR221. Insertion of the *ccdB* gene was confirmed by sequencing.

The cDNA library was cloned in the multi-copy prey plasmid pNXgate32 3HA (Obrdlik *et al.*, 2004). Before shuttling of the cDNA library, the *ccdB* gene was inserted into the prey vector to increase recombination efficiency, as described previously (Krügel *et al.*, 2012). The kanamycin resistance gene was still functional after insertion of the 737 bp toxin gene. Insertion was tested by restriction analysis and sequencing.

### Split ubiquitin screen

Large-scale yeast transformation was performed as described (Gietz and Woods, 2002). Yeast cells were transformed first with the bait construct.

The compound 3-Aminotriazol (3-AT) is a competitive inhibitor of the histidine biosynthesis *HIS3* gene product and, for each of the baits, the specific 3-AT concentration in the selection medium had to be determined to exclude autoactivation of the *HIS3* gene (Joung *et al.*, 2000). Titration of the 3-AT concentration was performed by co-expression of the bait construct with the empty pNubGgate. The 3-AT concentration required to prevent autoactivation was determined to be 40 mM for StSUT4.

Large-scale yeast transformation was performed according to the above-mentioned protocol; cells were resuspended in 10 ml distilled water and 500 μl cell suspension was plated on selection medium, without trp, leu, his, and 40 mM 3-AT. In order to determine the transformation rate, 4 μl of cells were plated on selection medium without trp and leu (SD-LT), and the number of primary transformants was counted after 3–4 d.

A saturated screen of the cDNA library (with 38 000 genes expected for *Solanum tuberosum*) was performed with a number of primary transformants of 10^6^ (number of independent yeast colonies carrying both plasmids and growing on medium -trp-leu) as described before (Krügel *et al.*, 2012). Sixty yeast colonies growing on selective medium (–leu –trp –ura –his) were selected for further analysis, and after restriction analysis, 23 restriction groups were identified. Fifteen different inserts were sequenced and 10 candidates showed the correct reading frame.

### Cloning of BiFC constructs

All other constructs were cloned using GATEWAY technology into VYNE and VYCE (Gehl *et al.*, 2009) allowing C-terminal fusion to the two halves of the YFP molecule (see primer list in Supplementary Table S1).

Sub-cellular localization of StSUT4 was performed by cloning the coding region into pK7YWG2.0 (Karimi, 2005) by GATEWAY technology using the primers for GATEWAY cloning mentioned above.

### Cloning of interaction partners in yeast expression vectors

Full length cDNAs from ETR2 and PCM1 were amplified from SUS vectors generated above using specific primers (Supplementary Table S1), and ligated into the yeast expression vector 112A1NE (Reinders *et al.*, 2002) after digestion with *Eco*RI and *Bam*HI. Constructs were checked by restriction analysis and sequencing. Co-expression in yeast was performed using different amino acid auxotrophies (112A1NE: trp-selection, pDR196: ura selection).

### Infiltration of tobacco leaves

Transient transformation of leaves from *N. benthamiana* with Agrobacterium suspension transformed with BiFC vectors was carried out as described previously (Krügel *et al.*, 2012). The Agrobacterium cultures with an OD_600_ ~0.1 were harvested and resuspended in infiltration buffer (10 mM magnesium chloride, 10 mM MES pH 5.7, 100 μΜ acetosyringone).

After 2 h incubation, the suspension was injected into the lower side of the leaves. The leaves were analysed the 3rd and 4th d after infiltration using a Zeiss LSM800 with Airyscan (Zeiss, Jena, Germany). The detection settings were chosen according to the fluorophores. Excitation of YFP was at 488 nm, and for chlorophyll, was at 640 nm.

### RNA quantification by qPCR

RNA extraction from source leaf tissue was performed with Trisure (Bioline, Luckenwalde, Germany) or peqGold Trifast (Peqlab, Erlangen, Germany) according to the manufacturer’s protocol. Reverse transcription was performed with the Qiagen Omniscript RT Kit according to the manual. Optimized conditions included using oligo(dT) primers for the initial reverse transcription reaction on approximately 1 µg of total RNA after digestion with RNase-free DNase (Qiagen, Hilden, Germany).

Aliquots of 0.2 µl of the 10 µl RT-reaction were used for the subsequent PCR reaction in the presence of SYBR Green with HotGoldStar DNA-Polymerase (Eurogentec, Seraing, Belgium) in a BioRad CFX Cycler using the CFX System Software (BioRad, Germany) using the following programme: denaturation at 95 °C for 30 s, annealing for 30 s at 61 °C, and elongation for 30 s at 72 °C, in a program of 45 cycles, in a 10 µl reaction volume. Primers were designed to obtain a 50–150 bp amplicon using Primer3 software (https://primer3.ut.ee/)). Ubiquitin was used as a reference gene and relative transcript abundance of the target gene was calculated with respect to reference gene using the delta Ct method (2^(-ΔΔCt)^). All primers used for real-time PCR are listed in Supplementary Table S1.

### Esculin uptake measurements

A sucrose transporter assay using esculin was performed exactly as described previously (Gora *et al.*, 2012). Yeast cells expressing StSUT1, StSUT4 or mutated versions were harvested by centrifugation at 1300 ×*g* and esculin (Merck, Darmstadt, Germany) at 1, 2, and 8 mM was added to 200 μl of phosphate buffer (25 mM Na_2_HPO_4_, pH 3, 5, or higher, as indicated in each figure) to each well. Uptake experiments under acidic pH conditions were performed in the presence of 25 mM citric acid buffer (4.275 g l^-1^ of sodium citrate dihydrate and 2.011 g l^-1^ of citric acid, pH 5.0). The incubation time was between 10 and 60 min. Competition experiments were conducted in the presence of 20 mM of sucrose, glucose, or sorbitol. The plates were vortexed for 30 s and incubated at 30 °C for 1 h while shaking. The plates were centrifuged at 1300 × *g* for 5 min and the supernatant discarded. Cells were washed by adding 200 μl of phosphate buffer to each well. Cells were resuspended by vortexing. The washing step was repeated, and the content of microtiter plates then transferred to a black microtiter plate for fluorescence reading in a spectrofluorometer, with excitation at 367 nm and emission at 454 nm. Relative fluorescence was determined in relation to cell density measurement at OD_600_ as described previously (Gora *et al.*, 2012).

## Results

### Members of the SUT4 sub-family show dual targeting to the vacuole and the plasma membrane

**Fig. 1.**
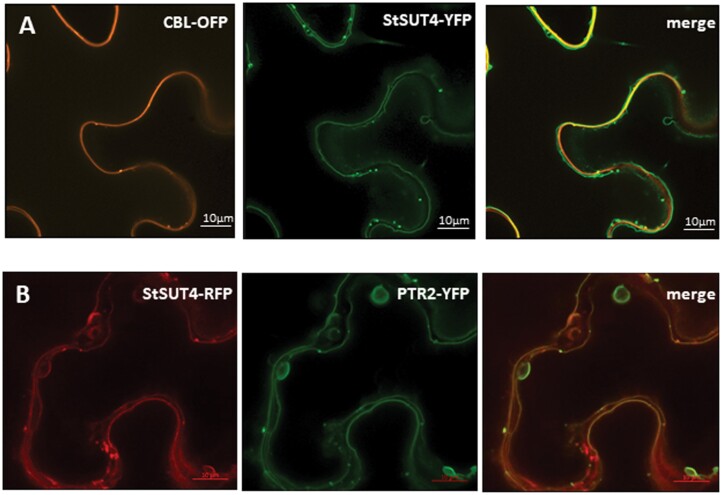
Sub-cellular localization of StSUT4. (A) Co-expression of a fluorescent StSUT4-YFP fusion protein (green) with a plasma membrane marker protein, CBL-OFP (orange) reveals no co-localization at the plasma membrane as previously observed under standard conditions (Garg *et al.* 2020). (B) StSUT4-RFP (red) is partially co-localized with a vacuolar marker protein, PTR2-YFP (green). Photographs were taken 3 d after infiltration with the Airyscan detector. Scale bar represents 10 µm.

As known for many other sucrose transporters of the SUT4 family, the StSUT4 transporter, although functional at the plasma membrane, does not always co-localize with the plasma membrane marker, but rather with vacuolar markers (Fig. 1; Chincinska *et al.*, 2013; Ho *et al.*, 2021). In bimolecular complementation assays (BiFC) showing the capacity of StSUT4 to homodimerize, the sub-cellular localization of the StSUT4 dimer changes evidently compared with its monomeric form (Garg *et al.*, 2020).

The sub-cellular localization of plant sucrose transporters is highly dynamic and affected by various external factors such as pH, temperature, oligomerization, ions, the substrate sucrose, inhibitors such as Brefeldin A (BFA) or cycloheximide (CHX), and biotic interactions such as colonization by mycorrhizal fungi, etc. (Krügel *et al*., 2008, 2012; Liesche *et al.*, 2008, 2010; Bitterlich *et al.*, 2014; Garg *et al.*, 2020; Hansch *et al.*, 2020). Sub-cellular localization of sucrose transporter might not only be affected by homo-oligomerization, but also by other interaction partners. With the aim to learn more about these post-translational effectors, it was obvious to screen for further interacting proteins.

### Identification of StSUT4-interacting candidates using the split ubiquitin system

**Fig. 2.**
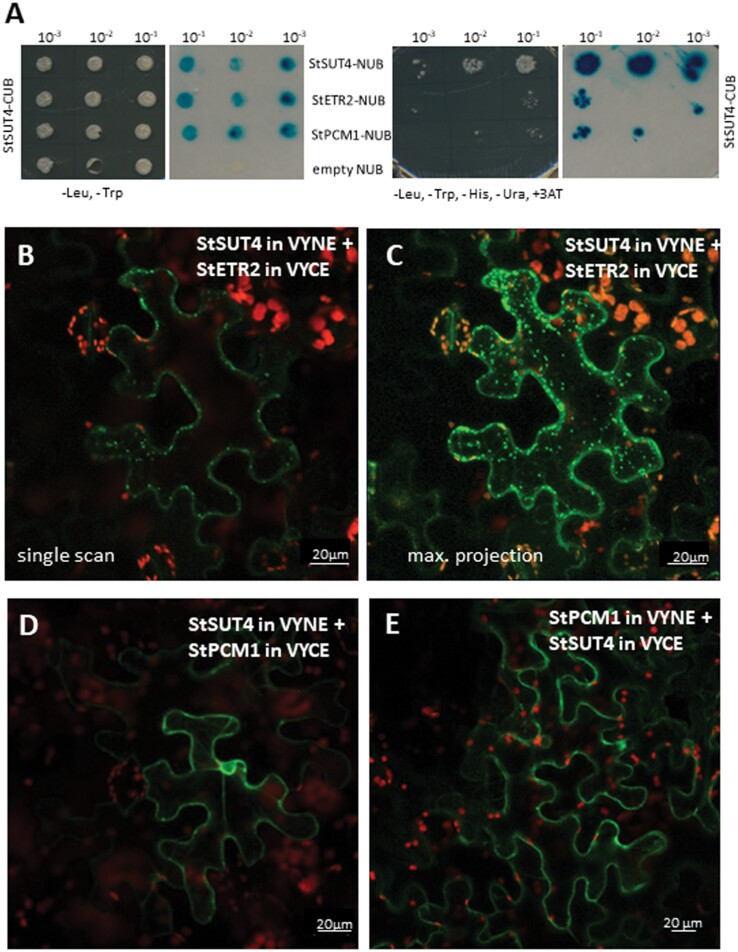
StSUT4 protein interactions. (A) Confirmation of interaction of full length cDNAs of StETR2 and StPCM1 with the sucrose transporter StSUT4 by the split ubiquitin system; and (B-E) BiFC analysis. (A) Homodimer formation between StSUT4 in Nub and StSUT4 in Cub was taken as positive control. Full length cDNAs of ETR2 and PCP1 enable yeast strain L40ccU to grow on selective medium. Quantification of interaction strength was performed using ß-glucuronidase assay (blue colour). (B) Confirmation of StSUT4-ETR2 interaction in BiFC experiments. Green colour indicates YFP reconstitution and successful interaction. Autofluorescence of chlorophyll is shown in red. A single scan is shown. (C) Same cell as shown in (B) in a maximum projection of a z-stack, showing interaction in intracellular compartments. (D, E) Confirmation of StSUT4 interacting with StPCM1 was performed by BiFC in both orientations. Interaction takes place close to the plasma membrane. Scale bars represent 20 μm.

In a saturated split ubiquitin screen of a potato cDNA library (Krügel *et al.*, 2012) with 10^6^ primary transformants using the full length *StSUT4* cDNA as a bait construct, 60 yeast colonies were found to be growing under highly stringent conditions (in the presence of 40 mM 3-AT). Restriction analysis helped to group them into distinct restriction groups, and sequencing identified 11 different cDNA inserts (Table 1). The full length cDNAs were cloned and interaction with StSUT4 was confirmed (Fig. 2A). The majority of the candidates represented integral membrane proteins, whereas four interacting candidates were predicted to encode soluble proteins (Table 1). Some of the candidates have been isolated several times independently. One ER-localized protein disulfide isomerase was identified previously to also interact with other plant sucrose transporters: StSUT1 (Krügel *et al.*, 2012) and SlSUT2 (Bitterlich *et al.*, 2014).

**Table 1.** StSUT4-interacting proteins identified by the split ubiquitin system using full length StSUT4 cDNA as a bait.

Name	Accession number	EST (*S. tuberosum*)	Predicted cellular compartment	Assumed function	TMDs
**Ethylene receptor 2 (ETR2)**	AY600436	423729	ER	Ethylene receptor	3
**Carboxylesterase 12** (**CXE12)**	XM006346397.2	EST600820 cDNA clone STMCZ57	Nucleus Cytosol	Alpha/beta hydrolase, thioesterase, lipase, interacts with DELLA	0-1
**calcium-load activated calcium channel** **(CLAC channel)**	NP_001275630	CV496516.1	ER	Calcium-selective channel required to prevent calcium stores from overfilling; ER calcium homeostasis	2-3
**Pectate lyase** **(PLL8)**	X1599	EST339302 (*S. lycopersicum*)	Cell wall	Pectate lyase	1
**Pectate lyase**	BG140571	EST481013 (*S. pennellii*)	Cell wall	Pectate lyase	1
**Photosystem II protein Y (PsbY)**	P80470	PPCAM72 PPCCQ17	Chloroplast, nuclear encoded	MYB TF, ethylene response	4
**Limb Development Membrane Protein 1 (LMBR1)-like ATPase**	XM550585 XP480815	AJ785414	PM?	Ubiquitin catabolism, thioesterase, cys endopeptidase, zinc ion binding	2
**Potato calmodulin 1 (StPCM1)**	J04559	47242.1	cytosol	Calcium binding	none
**Protein disulfide isomerase StPDI1**	DQ222488	Clone 098B03	ER	Chaperone, protein folding, quality control, escort	none
**Cathepsin D inhibitor**	DQ168325	cDNA clone 134F08	Cell wall	Kunitz-type proteinase inhibitor, Lysosomal Asp-protease Inhibitor	none (0-1)
**Unknown protein**	CP055238.1	cDNA clone cPR018C17 EST560871	unknown	Nodulin-like stress-induced protein, zinc finger protein	none

TMDs: number of predicted transmembrane domains.

EST: expressed sequence tag

ESTs are available from *Solanum tuberosum* according to the NCBI database, for most of the 11 interaction partners. For further investigation the most interesting was the interaction between the sucrose transporter StSUT4 and the ethylene receptor ETR2 belonging to the ETR family of membrane receptors, which is predicted to be localized in the ER membrane (Ju and Chang, 2012). SUT4 and ETR2 from tomato are tightly co-expressed according to various co-expression databases (TomExpress (http://tomexpress.toulouse.inra.fr/) ATTED II (https://atted.jp/); Supplementary Fig. S1). Another relevant interaction partner is the calcium binding protein StPCM1 (Takezawa *et al.*, 1995).

#### StSUT4 interacts with ETR2 in intracellular compartments

Bimolecular fluorescence complementation (BiFC) was used to confirm the interaction between StSUT4 and the ethylene receptor protein StETR2 identified in the split ubiquitin screen, using the full-length cDNA of StSUT4 as a bait (Fig. 2A). StETR2 is a 753 amino acid transmembrane protein with three transmembrane domains predicted to be integrated in the ER membrane, with the C-terminus orientated towards the cytoplasm and the N-terminus within the ER lumen (http://aramemnon.uni-koeln.de/). The structure of ETR2 is reminiscent to bacterial two-component systems with a N-terminal sensor domain able to bind copper ions and the C-terminal (cytoplasmic) histidine kinase domain (ethylene binding domain, GAF domain, kinase domain, receiver domain). Copper ions are needed for homodimer formation of ethylene receptor proteins to turn the complex into its active form (Ju and Chang, 2012). Further downstream signalling components involve CONSTITUTIVE TRIPLE RESPONSE1 (CTR1) Raf-like protein kinase and the transcription factor ETHYLENE INSENSITIVE 3 (EIN3; Ju and Chang, 2012).

Although ETR2 is predicted to be in the ER (Ju and Chang, 2012) and StSUT4 at the plasma membrane and the tonoplast, a clear interaction between StSUT4 and the StETR2 protein was confirmed by BiFC experiments; fluorescent YFP reconstitution was mainly observed in intracellular vesicles (Fig. 2B, C) and additionally in the cell periphery. All interaction studies were repeated in yeast using full length cDNAs cloned into the Nub and Cub vectors, and co-expressed with the StSUT4 cDNA in the Nub and Cub vector in various combinations (Supplementary Fig. S2).

#### StSUT4 interacts with PCM1 in the cell periphery

Another interesting StSUT4-interacting protein, StPCM1, identified by the split ubiquitin screen, was also confirmed in BiFC experiments (Fig. 2D, E; Supplementary Fig. S3). Here, a completely different localization of the heteromeric complex was observed: StPCM1 and StSUT4 interacted with each other mainly in the cytosol close to the plasma membrane. Since PCM1 is a calcium-binding protein with four calcium binding motifs, the question arose whether calcium ions were able to somehow affect StSUT4 activity, localization, dimerization, or protein-protein interactions. In order to test these hypotheses, we first needed to establish a functional assay, since StSUT4 transports sucrose with low affinity and at a very low rate. This is the reason why functional characterization of StSUT4 transport kinetics was performed only in yeast but not in *Xenopus* oocytes (Weise *et al.*, 2000).

### Establishment of a functional assay using esculin as sucrose analogue

Esculin is a fluorescent coumarin-derivative that is well established to serve as an equivalent sucrose analogue in uptake experiments with type I plant sucrose transporters, and also for dicotyledonous, but not monocotyledonous type II transporters (Gora *et al.*, 2012). Thus, we had to first test whether or not the fluorescent esculin assay was suitable for SUT4-clade (or type III-like) sucrose transporters as well.

**Fig. 3.**
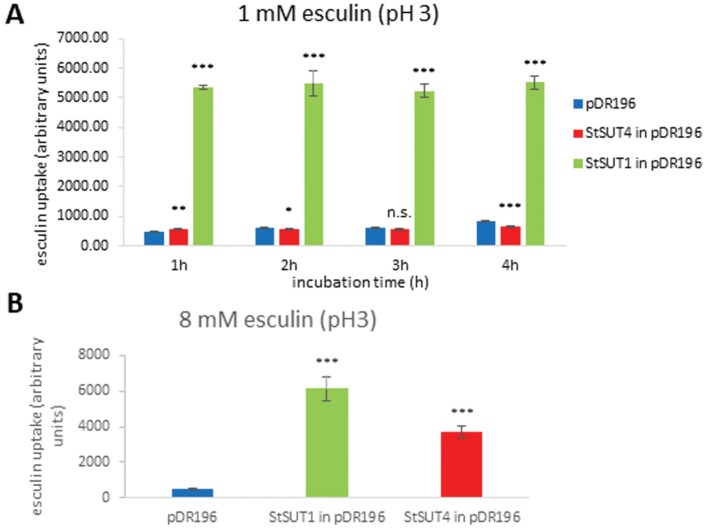
Esculin uptake assay. Establishment of a functional assay for StSUT4 in yeast cells using esculin as fluorescent sucrose analogue and determination of the optimal substrate concentration. (A) StSUT1, but not StSUT4 shows significantly higher esculin uptake at 1 mM substrate concentration than the empty vector control (pDR196). (B) StSUT4 and StSUT1 show significantly higher esculin uptake than the empty vector control (pDR196) at 8 mM substrate concentration after 1 h of incubation. Data are means ±SD; *n*=3. Student’s *t*-test was performed (*** *P*<0.001, **P<0.01 and *P<0.05).

The Michaelis Menten constant (K_M_) for StSUT1 is close to 1 mM sucrose, and 1 mM of esculin was suitable for StSUT1-mediated sucrose uptake measurements (Fig. 3A). StSUT4, however, did not show sucrose uptake using 1 mM of esculin (Fig. 3A). The affinity of SUT4-clade sucrose transporters towards the substrate sucrose was much lower than for SUT1 (Weise *et al.*, 2000; Weschke *et al.*, 2000). Therefore, we tested increasing substrate concentrations (2 mM, up to 8 mM) and were successful in establishing a functional uptake assay using increased esculin concentrations (Fig. 3B).

**Fig. 4.**
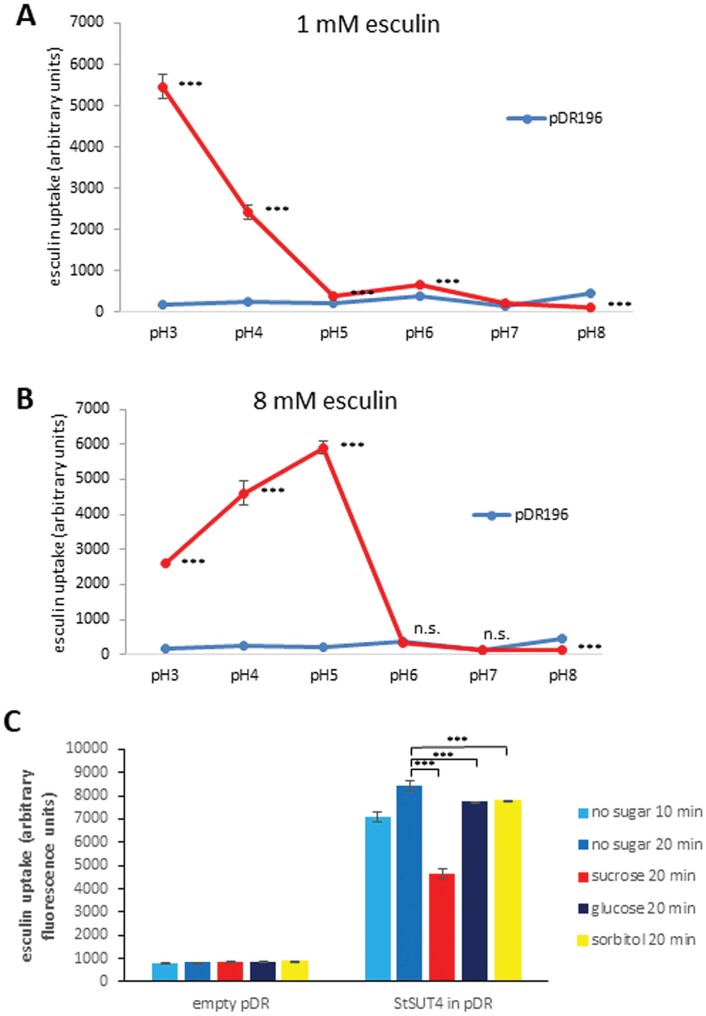
pH dependence of esculin uptake by StSUT1 and StSUT4 in yeast cells. (A) StSUT1 shows a pH optimum in the highly acidic range (pH 3). (B) StSUT4 not only shows a reduced affinity towards its substrate but also a different pH dependence than StSUT1 with a pH optimum at pH 5. Data are means ±SD; *n*=4 independent measurements. (C) StSUT4-mediated esculin uptake in yeast cells in the presence of unlabelled sugars or no sugar. Competition studies revealed specificity of esculin uptake mediated by StSUT4 that is efficiently down-regulated in the presence of 20 mM sucrose. Data are shown as means ±SD, *n*=9 with three biological and three technical replicates, Student‘s *t*-test was performed (***P<0.001).

Increasing the esculin concentration to 8 mM revealed a clear StSUT4-mediated esculin uptake that was significantly higher than the empty vector control (*P*<0.001). Thereby, the low affinity of StSUT4 towards the substrate sucrose or esculin was confirmed. Not only did the substrate affinity differ between SUT1 and SUT4 transporters, but pH dependence was different as well. Esculin uptake was higher at pH 5 than at pH 3 (Fig. 4B) which was also observed in previous investigations using ^14^C-sucrose uptake experiments (Weise *et al.* 2000). This is in contrast to StSUT1 with a pH optimum in the highly acidic range (Fig. 4A; Krügel *et al.*, 2008). The extremely strong increase in sucrose uptake at very low pH as previously observed for StSUT1 and ZmSUT1 (Krügel *et al.* 2008) was not observed for StSUT4, neither in case of ^14^C-sucrose (Weise *et al.* 2000), nor for esculin uptake (Fig. 4B). pH-dependent esculin uptake was also confirmed by confocal microscopy using UV excitation (Supplementary Fig. S4A). Following this, all sucrose uptake experiments using StSUT4 were performed at optimal conditions with 8 mM esculin and at pH 5.0.

The specificity of StSUT4-mediated esculin uptake was then tested under pH 5.0 in the presence of 8 mM of esculin and an excess of unlabelled sugars (Fig. 4C). Whereas addition of 20 mM sucrose decreased the StSUT4-mediated esculin uptake by almost 50%, the addition of an excess of 20 mM glucose or sorbitol decreased it by only 10 % (Fig. 4C).

**Fig. 5.**
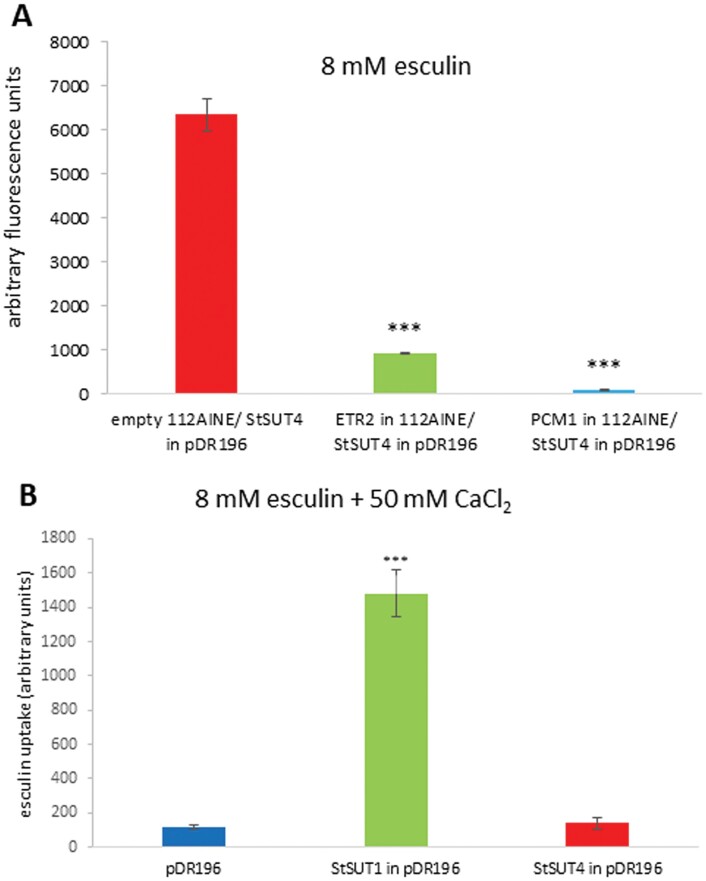
(A) StSUT4-mediated esculin uptake in yeast cells in the presence or absence of interaction partners. Co-expression of StSUT4-interaction partners StETR2 and StPCM1 reduces StSUT4-mediated esculin uptake. StSUT4 in pDR196 was co-expressed with the empty vector 112A1NE (red), StETR2 in 112A1NE (green) or StPCM1 in 112A1NE (blue). Data are means ±SD. Uptake was measured at pH 5. (B) Both sucrose transporters, StSUT1 as well as StSUT4 are efficiently inhibited by addition of 50 mM CaCl_2_ after 1 h of incubation at pH 3. Student‘s *t*-test was performed (***P<0.001).

Under these optimal conditions, the presence of StSUT4-interacting proteins was tested when expressed in the same yeast cells (Fig. 5A). Both interacting proteins, the ethylene receptor ETR2, as well as the calcium-binding PCM1 protein, inhibited StSUT4-mediated esculin uptake significantly (*P*< 0.001; Fig. 5A), whereas co-expression of the empty vector did not.

### Calcium ions affect functionality, expression, dimerization and targeting of StSUT4

#### Calcium ions inhibit StSUT1- and StSUT4-mediated uptake of esculin

As previously observed in ^14^C-sucrose uptake experiments using StSUT1 (Krügel *et al.*, 2013), the activity of StSUT4 was drastically reduced in the presence of divalent cations such as calcium ions (Fig. 5B). The inhibitory effect of calcium ions on StSUT1, as well as StSUT4-mediated esculin uptake was confirmed by confocal microscopy using UV excitation (Supplementary Fig. S4B). This decrease in activity was postulated to be due to the removal of the transporter from the plasma membrane via endocytosis. Another possibility is that the expression strength or protein stability is negatively affected by increasing calcium concentrations.

#### Calcium (and magnesium) ions induce expression of StSUT4

**Fig. 6.**
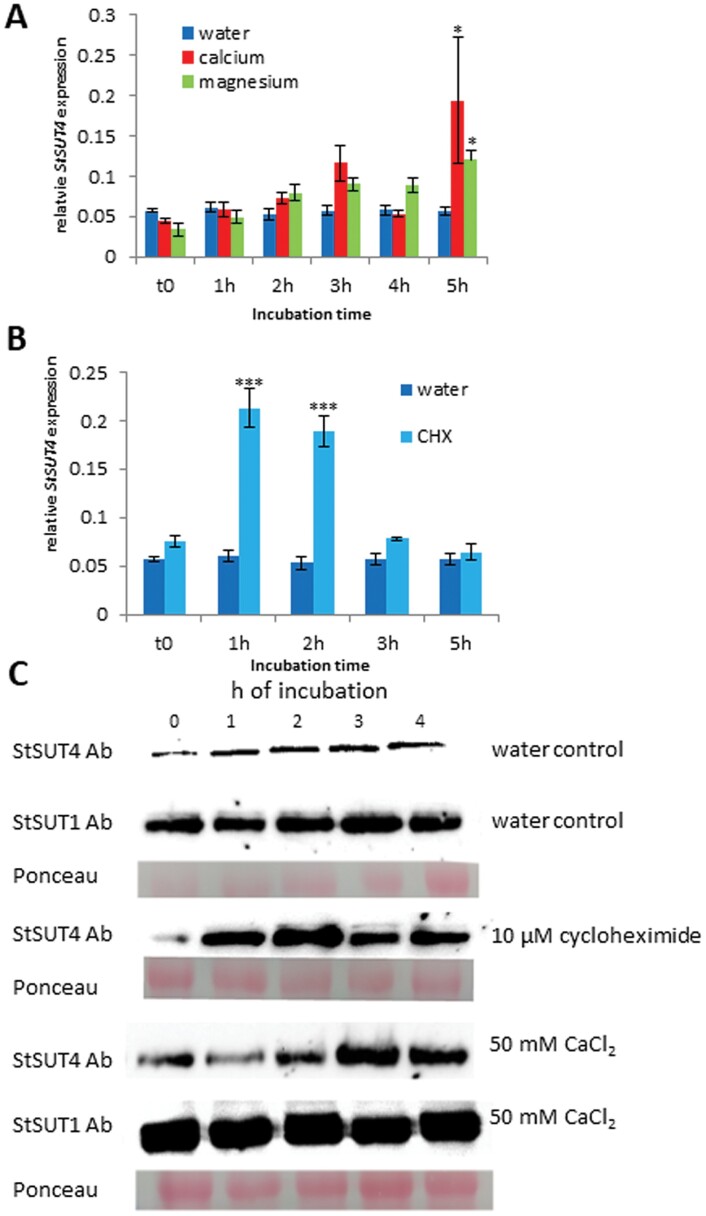
Quantification of *StSUT4* transcripts and protein accumulation in response to various inhibitors or effectors. (A) Quantification of *StSUT4* transcript amount via qPCR analysis using ubiquitin as a reference gene after treatment of source leaf material with either 50 mM CaCl_2_, 50 mM MgCl_2_ or water. Plant material was harvested at different time points (0–5 h).(**B)** qPCR analysis of *StSUT4* transcript amount in source leaf material treated with the translational inhibitor cycloheximide (10 µM) for the indicated period of time (0–5 h) show a transient increase in transcript accumulation. Student’s *t*-test was performed to obtain significance values (*n*=4; ***P<0.001, **P<0.01, and *P<0.05). Ab: antibody. (**C)** Western blot analysis using the microsomal fraction of potato leaves incubated for several hours in water (water control), 10 µM of cycloheximide, or 50 mM CaCl_2_. Incubation for several hours in 50 mM CaCl_2_ show increased protein amount of StSUT4, but unchanged levels of StSUT1 protein. Cycloheximide effects on StSUT1 were published earlier (Kühn *et al.*, 1997).

In order to investigate whether reduced expression or protein abundance could be the reason for reduced StSUT4-mediated esculin uptake, the transcript accumulation and protein abundance was measured. Increasing *StSUT4* transcript accumulation in the presence of calcium or magnesium ions compared with the water-treated control (Fig. 6A) helped to exclude that decreased expression might be the reason for reduced esculin uptake in the presence of increasing calcium (Fig. 5B).

To test protein stability, western blots using the microsomal fraction of water, cycloheximide (CHX), or calcium chloride-treated potato leaves were performed (Fig. 6C). StSUT1 half-life in the presence of CHX was investigated earlier (Kühn *et al.*, 1997). Usage of a specific affinity-purified StSUT4 peptide antibody (Weise *et al.*, 2000) revealed a short protein half-life of StSUT4 in the presence of the translational inhibitor CHX (Fig. 6C), and a rather stabilizing effect in the presence of calcium chloride (Fig. 6C). No increase in the protein amount was detectable for StSUT1 (Fig. 6C). Therefore, a reduced protein stability can also be excluded as the reason for reduced uptake. A calcium-dependent regulation of StSUT4 at the post-translational level was more likely.

#### StSUT4 is regulated at the transcriptional and post-transcriptional level

Far-red light increases *StSUT4* transcript abundance and *StSUT4*-RNAi plants do not show shade avoidance response and seem to be far-red light insensitive (Chincinska *et al.*, 2008). Accumulation of *StSUT4* mRNA is increased under far-red light enrichment, as is the case under canopy shade (Chincinska *et al.*, 2008). This increase in *StSUT4* transcript accumulation is under control of phytochrome B, given that in *phyB* antisense plants no such far-red light dependence is observed (Liesche *et al.*, 2011). This increased accumulation is most likely due to increased transcript half-life, because in the presence of transcriptional inhibitors such as actinomycin D, by inhibiting *de novo* synthesis of *StSUT4* transcripts, the degradation of this specific mRNA is delayed, as shown by quantitative qPCR (Liesche *et al.*, 2011). Both observations suggest a tight control of *StSUT4* transcript stability at the post-transcriptional level. Potentially, this permanent transcript degradation occurs under involvement of a far-red light sensitive photoreceptor such as phyB.

It was known from previous investigations that the half-life of *StSUT2* and *StSUT4* transcripts is also prolonged in the presence of cycloheximide (CHX), an inhibitor of translation (He *et al.*, 2008; Fig. 6B. This suggests that very short-lived ribonucleases are engaged in continuous transcript degradation. This transient increase in *StSUT4* transcript accumulation (Fig. 6B) has consequences on the protein amount, as seen in western blots in the presence of CHX (Fig. 6C).

#### Calcium ions increase internalization of StSUT4 (but not of StSUT1)

**Fig. 7.**
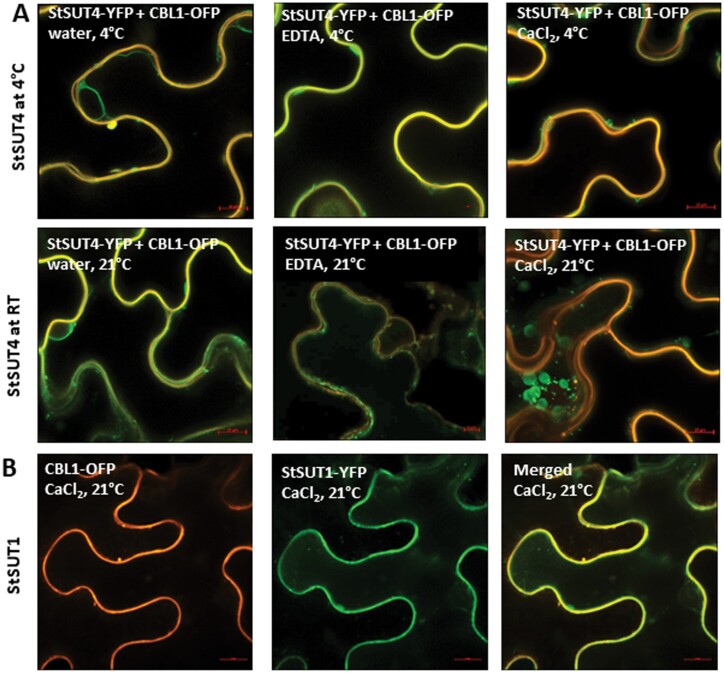
Changes in sub-cellular localization of StSUT1 and StSUT4 in response to CaCl_2_ treatment. (A) Overnight treatment with 50 mM CaCl_2_ at 21 °C induces increased vesicle formation of StSTUT4-YFP (shown in green) in the intracellular lumen of *Nicotiana benthamiana* epidermis cells, whereas water or EDTA do not. These effects are visible to a smaller extent if leaves were incubated in the cold (4 °C, upper row). YFP constructs (shown in green) were co-infiltrated with a PM marker (shown in orange) as before (Garg *et al.* 2020). (B) No internalization of StSUT1-YFP (green) from the plasma membrane was observed in response to overnight incubation of infiltrated leaves in 50 mM CaCl_2_ at 21 °C. StSUT1 remains co-localized with the PM marker protein (orange) even in the presence of high amounts of calcium. Images were taken 3-4 d after infiltration using the Airyscan detector after the indicated time of incubation. Scale bar represents 10 μm.

As shown previously using confocal microscopy, calcium ions were able to specifically induce internalization of StSUT4 protein (Fig. 7A), whereas no such effect was observed for StSUT1 (Fig. 7B), a high affinity sucrose transporter that is also able to interact with StPCM1 (Supplementary Fig. S2). These effects are time- and temperature-dependent. The effects were more pronounced at prolonged incubation (overnight) with the different effectors (CaCl_2_ or EDTA), and less pronounced at low temperature (4 °C; Fig. 7A).

Thus, not only homodimer formation, but also calcium ions affect sub-cellular targeting of StSUT4. The next question was whether or not calcium also affects the dimerization behaviour of StSUT4, and thereby indirectly, its sub-cellular localization.

#### Calcium inhibits StSUT4 homodimerization and activity

To investigate the role of calcium in regulating StSUT4 dimerization, split ubiquitin experiments were performed in yeast in the presence of increasing calcium concentrations (Supplementary Fig. S5). Whereas EDTA, as well as magnesium at high concentration impaired normal yeast growth, calcium ions were not harmful for normal yeast growth (Rees and Stewart, 1997). However, a clear inhibitory effect on homodimer formation was observed in the split ubiquitin system, and quantified with increasing amount of calcium (Supplementary Fig. S5). The question remains, whether the detrimental effect of calcium on StSUT4 activity is due to its increased internalization, its reduced dimerization, or both.

#### Site-directed mutagenesis of a diacidic motif inhibits transport activity

A highly conserved diacidic motif is present in all known sucrose transporters, and is localized in the seventh transmembrane spanning domain of all transporters. This motif represents a putative binding site of positively charged ions and seems to be crucial for sucrose transporter activities (Sun *et al.*, 2012; Krügel *et al.*, 2013).

We used site-directed mutagenesis of this highly conserved diacidic motif (D_304_T_305_D_306_) in order to dissect out the ability of plant sucrose transporters to dimerize, to be internalized, and their functionality.

**Fig. 8.**
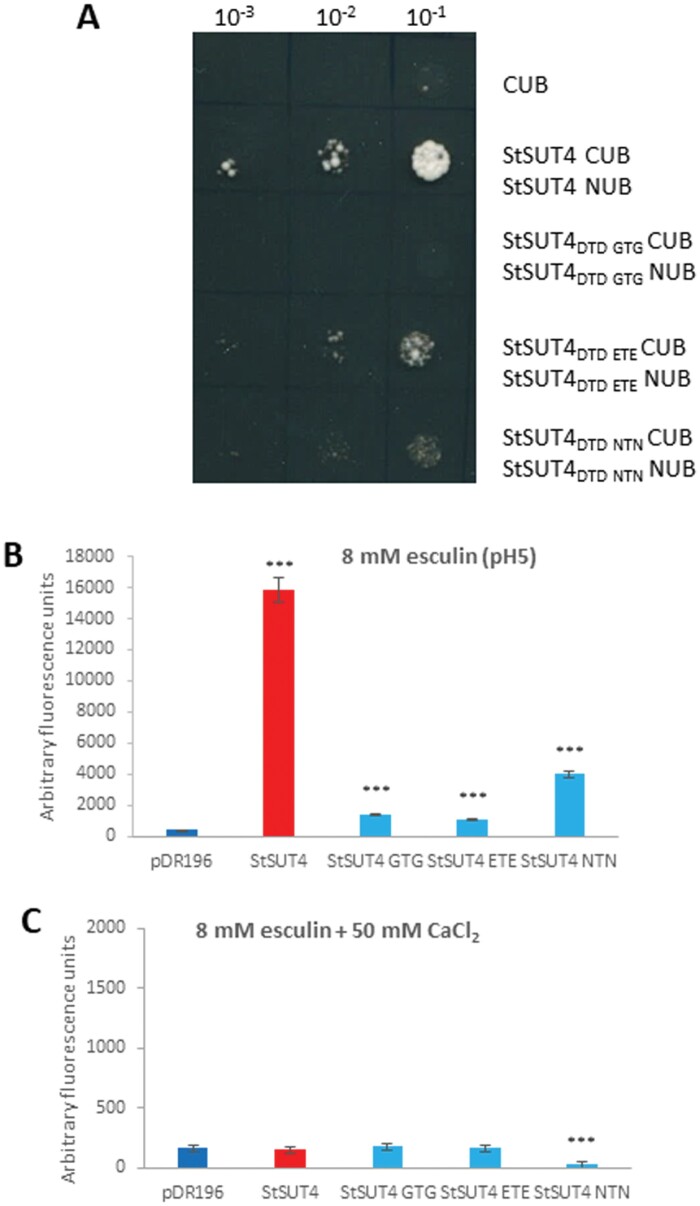
(**A)** Split ubiquitin system with mutagenized StSUT4 constructs where the highly conserved DTD motif within the seventh transmembrane spanning domain is replaced by either GTG, NTN, or ETE, show the importance of this motif for efficient homodimerization. (**B)** StSUT4-mediated esculin uptake after mutagenesis of the conserved DTD motif at optimal pH (pH 5) and substrate concentration (8 mM). Mutagenesis of the DTD motif strongly affects StSUT4 transport activity. (**C)** Neither StSUT4, nor the DTD mutant constructs of StSUT4 are functional in the presence of 50 mM CaCl_2_. Incubation time was 60 min. Note the difference in scale. Student’s *t*-test was performed to obtain significance values (*n*=4; ***P<0.001, **P<0.01, and *P<0.05).

Interestingly, the mutagenesis of this motif in StSUT4 also affects dimerization of the transporter, and only replacement of the two aspartic acid residues by glutamic acid residues seemed to allow residual dimer formation, arguing for a charge-dependence of the homodimer formation (Fig. 8A). Replacement of the two aspartic acid residues by uncharged amino acids such as glycine or asparagine completely disturbed homodimer formation (Fig. 8A).

Regarding sucrose transport activity, this DTD motif is crucial, since its mutagenesis reduced esculin uptake by almost 90% (Fig. 8B). The inhibitory effect of calcium ions on the uptake behaviour of StSUT4 at an optimal of pH 5 was also detectable for the mutant constructs, indicating that homodimer formation alone was not responsible for internalization, and that the DTD motif does not represent a calcium binding domain (Fig. 8C). It can be assumed that homodimerization is not a pre-requisite for internalization.

Calcium-mediated inhibition of sucrose uptake does not require the highly conserved D_304_T_305_D_306_ motif that is essential for sucrose transport activity of StSUT1 as well as of StSUT4. Further investigations will be required to understand how the inhibitory effect by divalent cations is achieved.

**Fig. 9.**
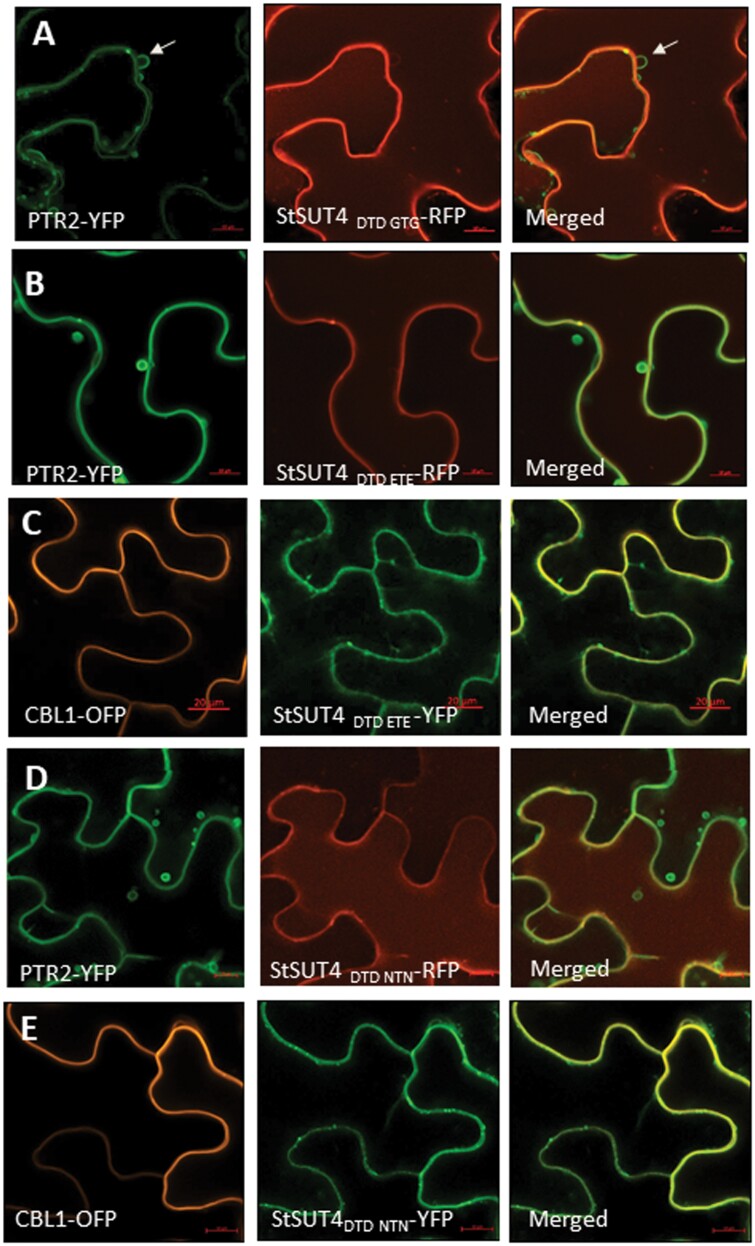
Sub-cellular localization of StSU4 mutants. Co-localization experiments with StSUT4 mutant constructs co-infiltrated with either vacuolar (PTR2-YFP) or plasma membrane (CBL1-OFP) marker proteins. **(A, B, D)** None of the DTD mutant constructs could be co-localized with the vacuolar marker protein PTR2-YFP (arrow). **(C, E)** The StSUT4 DTD ETE-YFP and the StSUT4 DTD NTN-YFP construct show perfect overlap with the plasma membrane marker CBL1-OFP. Images were taken 3 d after infiltration using the Airyscan detector. Scale bars represent 10 µm (A, B, D, E) or 20 µm (C).

Sub-cellular localization of the mutant constructs of the highly conserved diacidic DTD motif suggests another function. Diacidic motifs within the C-terminal region of aquaporins have been described to be responsible for efficient ER export of homo- or heteromeric complexes (Zelazny *et al.*, 2009). Therefore, the sub-cellular localization of the SDM constructs were analysed in detail (Fig. 9). None of the mutants co-localized with the peptide transporter PTR2, that can be used as a vacuolar marker protein. Replacement of the DTD motif by either GTG, NTN or ETE enhanced plasma membrane targeting (Fig. 9).

In conclusion, the highly conserved DTD motif is required for functionality and dimerization of sucrose transporters, but not for calcium-mediated inhibition of sucrose transport capacity, since mutant constructs of StSUT4 with replacement of the two aspartic acid residues can still be inhibited by the supply of calcium chloride (Fig. 8B, C). Although the capacity to form homodimers seems to be reduced in the mutants, the efficiency of plasma membrane targeting is rather enhanced (Fig. 9).

## Discussion

### Characterization of StSUT4-mediated uptake

We have shown for the first time, that the fluorescent esculin uptake assay is not only useful for activity measurement of type I sucrose transporters (Gora *et al.*, 2012; Reinders *et al.*, 2012), but could also be established for a low affinity type III sucrose transporter of the SUT4 clade (Kühn and Grof, 2010) if the substrate concentration is adapted. Using this activity assay, it was possible to investigate further effectors on SUT4 transport activity, as well as confirm the pH dependence previously measured by ^14^C-sucrose transport assays (Weise *et al.*, 2000). Thereby, the strong difference not only in substrate affinity, but also pH dependence between SUT1 (type I) and SUT4 (type III) transporters could be elucidated (Fig. 4).

Furthermore, we showed that both transporters are efficiently inhibited by divalent cations such as calcium or magnesium (Fig. 5B; Krügel *et al.*, 2013). At least in the case of StSUT4, it can be excluded that this decrease in activity is due to a decreased expression or reduced protein abundance.

Interestingly, internalization to luminal vesicles can be induced by calcium treatment in the case of StSUT4, but not for StSUT1 (Fig. 7). Both homodimerization as well as calcium treatment seem to favour internalization of StSUT4. Since calcium in parallel impairs homodimer formation of StSUT4, it is concluded that homodimerization is not a pre-requisite for internalization.

Site directed mutagenesis of a highly conserved di-acidic motif within the seventh transmembrane spanning domain revealed an essential function for StSUT4 activity (Fig. 7B), as is was previously shown for other sucrose transporters such as OsSUT1 (Sun *et al.*, 2012) and StSUT1 (Krügel *et al.*, 2013). This motif seems to be also required for StSUT4 homodimer formation (Fig. 8A). But it is unlikely that it represents a putative calcium binding domain, since calcium-dependent inhibition of transport activity is still observed after replacement of this motif (Fig. 8C).

### StSUT4 is co-expressed and interacts with StETR2

Ethylene, as well as gibberellic acid and auxin are involved in the shade avoidance response of higher plants (Pierik *et al.*, 2004a). Phytochrome B (PhyB) is responsible for the perception of the red: far-red light ratio and triggers the shade avoidance response involving elongated internode growth, early flowering, and hyponastic leaf movement (Ballaré, 2017). It was shown in tobacco plants that an increase in far-red light is accompanied by an increase in ethylene production (Pierik *et al.*, 2004a), and that ethylene-insensitive tobacco plants show only a reduced shade avoidance response (Pierik *et al.*, 2004b). Transgenic potato plants with reduced *StSUT4* expression are also defective in the shade avoidance response (Chincinska *et al.*, 2008), and produce lower amounts of ethylene (Chincinska *et al.*, 2013). At the same, time the transcript amount of the key enzyme of ethylene biosynthesis, ACC oxidase, shows reduced levels in *StSUT4*-silenced potato plants (Chincinska *et al.*, 2008), suggesting that StSUT4 and ethylene biosynthesis are tightly linked to each other. Reduced ethylene biosynthesis in these plants could be the reason for the missing shade avoidance syndrome of *StSUT4*-RNAi plants (Chincinska *et al.*, 2008).

StSUT4 was shown to be important for the phyB-dependent shade avoidance syndrome of potato plants (Chincinska *et al*., 2008, 2013). *StSUT4* transcripts accumulate in response to increased far-red light enrichment under canopy shade in a phyB-dependent manner (Chincinska *et al.*, 2008). This increase in transcript abundance under shade conditions is not due to increased transcriptional activity, but rather due to increased transcript stability (Liesche *et al.*, 2011) and the photoreceptor PhyB seems to be involved in this increased transcript stability as seen in *phyB* antisense potato plants (Liesche *et al.*, 2011). Here again, it was confirmed that *StSUT4* transcript stability is tightly controlled post-transcriptionally not only in a light quality-dependent manner (Liesche *et al.*, 2011), but also by short lived proteins such as ribonucleases (Fig. 6A; He *et al.*, 2008)).

### Interactome of StSUT4

Elucidation of the StSUT4 interactome reveals the presence of several cell wall-localized interaction partners, among them, two different pectate lyases (Table 1). A recent study describes a strong link between ethylene signalling, calcium signalling, sugar transport and cell wall remodelling as an important target of ethylene perception, with respect to pollen tube growth in tomato plants (Althiab-Almasaud *et al.*, 2021). The authors identified the link via transcriptome analysis of *ETR* loss-of-function as well as gain-of-function mutants in tomato without describing the connection at the molecular level. The StSUT4-ETR2 interaction might represent a link as to how sucrose, calcium and ethylene signalling are interconnected by direct physical contact.

### Sucrose and ethylene are involved in the entrainment of the circadian clock

The ethylene receptor ETR2 is known to interact with itself and with ETR1, another ethylene receptor protein that is expressed in the phloem, and ERS1 (Grefen *et al.*, 2008). In tomato plants, SlSUT4 and ETR2 are co-expressed, according to the TomExpress database (Supplementary Fig. S1), and also in Arabidopsis, a link between the ethylene perception and sucrose metabolism is suggested by the co-expression of AtETR2 with the sucrose synthase AtSUS4 (according to ATTED II database).

A link between ethylene signalling and the sucrose status is also evident in the regulation of circadian genes: sucrose and ethylene are both involved in the entrainment of the circadian clock (Haydon *et al.*, 2017). It is suggested that the sugar-dependent entrainment of the circadian clock occurs via the transcription factor bZIP63 (Frank *et al.*, 2018). Sugars are required to adjust the phase of the circadian clock by repressing PSEUDO-RESPONSE REGULATOR (PRR7), an inhibitor of CIRCADIAN CLOCK-ASSOCIATED 1 (CCA1; Haydon *et al.*, 2013). It was shown by inhibition of photosynthesis, that endogenous oscillation of sugars represents a kind of metabolic feedback signal, that entrains and maintains circadian rhythms in Arabidopsis. This endogenous sugar oscillation entrains the circadian oscillator by repressing the morning-expressed gene *PRR7*. It is assumed that sugars repress the pseudo-response regulator PRR7 during the morning to adjust the phase of the circadian clock (Haydon *et al.*, 2013).

Furthermore, it is known that GIGANTEA (GI) is needed to sustain sucrose-dependent circadian rhythms in the dark. Herein, sucrose is supposed to stabilize the GI protein in darkness, thereby involving CTR1, a negative regulator of ethylene signalling that acts upstream of GI.

Ethylene shortens the circadian period, and sucrose can mask these negative effects of ethylene on the circadian system by stabilizing circadian oscillator proteins (Haydon *et al.*, 2017). A further ethylene signalling component, EIN3, is destabilized by sugar (Yanagisawa *et al.*, 2003).

From *StSUT4-*silenced potato plants we learned that the oscillation of sugars and its export from leaves is delayed in *StSUT4*-RNAi plants, suggesting that the entrainment of the circadian clock is disturbed (Chincinska *et al.*, 2008). Not only is the accumulation of soluble sugars and starch delayed in *StSUT4*-RNAi plants, but also the expression of the main phloem loader *StSUT1* that oscillates with lower amplitude when *StSUT4* is silenced (Supplementary Fig. S6A). In parallel, diurnal ethylene production is completely abolished in *StSUT4*-RNAi potato plants (Chincinska *et al.*, 2013). The transcript abundance of GI is also affected in *StSUT4*-silenced potato plants (Chincinska *et al.*, 2013). In this context it would be interesting to quantify GI protein stability. The quantification of relevant components of the ethylene signal transduction pathway via real time qPCR revealed up-regulation of EIN3 in in *StSUT4* overexpressing plants (Supplementary Fig. S6B). It remains to be answered whether overexpression of *StSUT4* causes a similar shift in sucrose oscillation in potato leaves leading to out of phase-oscillation of sucrose-responsive genes, as shown for Arabidopsis (Supplementary Fig. S7, S8).

In a number of species, the level of mature miR172 also changes during the day (Wang *et al.*, 2016). A previous investigation showed sucrose-inducibility of *miR172* transcript accumulation, changes of *miR172* transcript level during the day, and induction of *miR172* transcript levels in *StSUT4*-RNAi plants (Garg *et al.*, 2021). It is therefore assumed that *StSUT4* is involved in the maintenance and adjustment of the phase of the circadian clock via changes in sucrose oscillation. Out of phase oscillation of sucrose levels in the leaves of *StSUT4-*silenced plants might be responsible for increased levels of mature *miR172*, early flowering and tuberization under non-inductive photoperiodic conditions (summarized in Supplementary Fig. S7). It cannot be excluded that other circadian clock-dependent processes are also affected in these plants (Supplementary Fig. S8).

A very recent study identifies *StSUT4*, together with the sugar transporter *SUGARS WILL EVENTUALLY BE EXPORTED TRANSPORTER* (*SWEET11)* as core circadian clock genes in below-ground storage organs, showing a robust circadian transcriptional rhythm in detached tubers independent from leaves, with important implication for the regulation of tuberization in potato crop plants (Hoopes *et al.*, 2022). These transcriptomic data further strengthen the hypothesis of StSUT4 as a key component of the circadian clock in potato tubers.

## Supplementary data

The following supplementary data are available at *JXB* online.

Fig. S1. Co-expression of ETR2 and SUT4.

Fig. S2. Confirmation of interaction in yeast.

Fig. S3. StPCM1 interactions.

Fig. S4. Microscopic analysis of yeast cells after esculin incorporation.

Fig. S5. Homodimerization of StSUT4 in the presence of calcium.

Fig. S6. Expression analysis of *StSUT4*-RNAi plants.

Fig. S7. Hypothetical model illustrating the link between sucrose and ethylene signalling via StSUT4 and StETR2 interaction affecting the entrainment of the circadian clock.

Fig. S8. Hypothetical model illustrating the impact of sucrose in the entrainment of the circadian clock.

Table S1. Primers used in this study.

erac378_suppl_Supplementary_Figures_S1-S7

## Data Availability

All data supporting the findings of this study are available within this paper and within the supplementary material published online.
