# Supplementary material for: Elucidation of the interactome of the sucrose transporter StSUT4: sucrose transport is connected to ethylene and calcium signalling
Source: J Exp Bot. 2022 Sep 16;73(22):7401–16. doi: 10.1093/jxb/erac378 (PMC9730799; doi:10.1093/jxb/erac378)
Supplement: erac378_suppl_Supplementary_Figures_S1-S7 [file erac378_suppl_supplementary_figures_s1-s7.pdf]

Supplemental Fig. S1

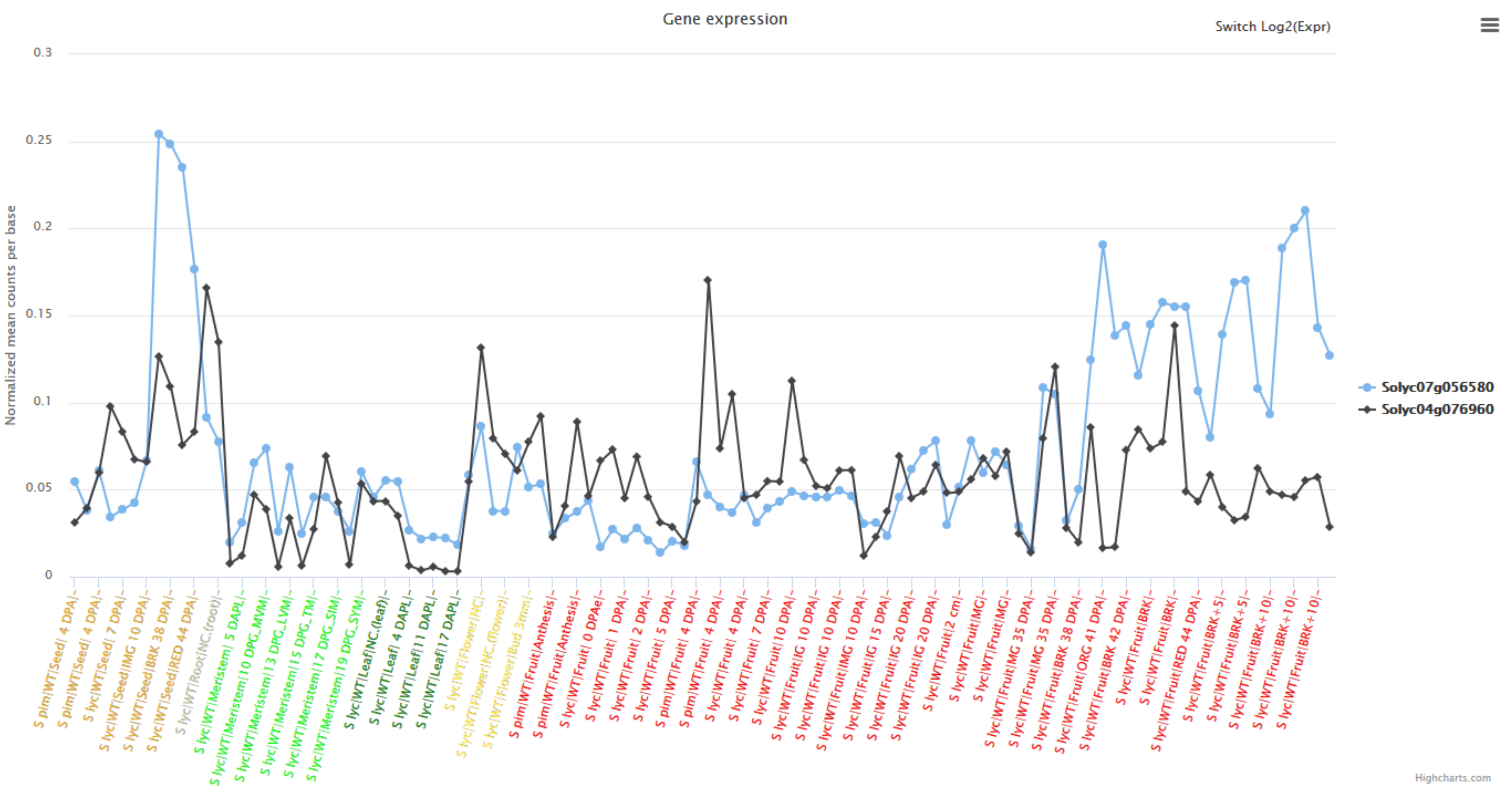

Supplemental Fig. S1. **Co-expression of ETR2 and SUT4**. According to co-expression databases such as the tomato TomExpress database (http://tomexpress.toulouse.inra.fr/) and ATTED II (https://atted.jp/), the two candidate genes SlSUT4 (Solyc04g076960) and the ethylene receptor SlETR2 (Solyc07g056580) are tightly co-expressed in almost all tissues supporting the assumption that SUT4 and ETR2 physically interact with each other as revealed by a split ubiquitin screen.

The Arabidopsis ETR2 gene is co-expressed with the sucrose synthase SUS4 gene (ATTED II database).

Supplemental Fig. S2

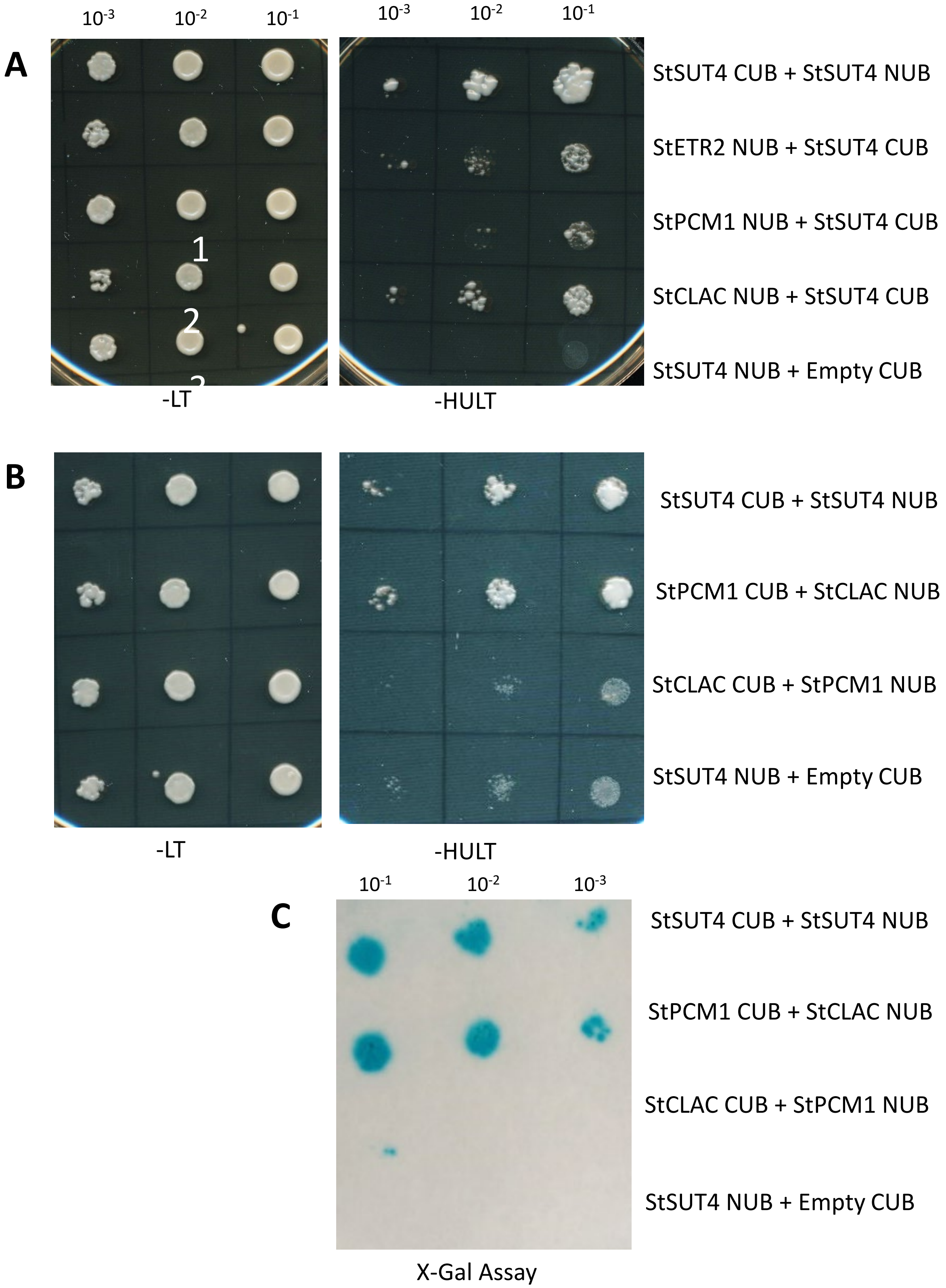

Supplemental Fig. S2 Confirmation of interaction in yeast. Full length cDNAs from three SUT4-interaction partners: ETR2, PCM1 and the CLAC channel are tested in a split ubiquitin assay. StSUT4 homodimer formation was used as a positive control. Yeast growth in the presence of the empty CUB vector was used as a negative control (**A**). Interaction between StSUT4 and the CLAC channel is unidirectional: if the CLAC channel is cloned into CUB, no interaction can be detected (**B**). Quantification of interaction strength was performed using X-gal assays (**C**).

Supplemental Fig. S3A

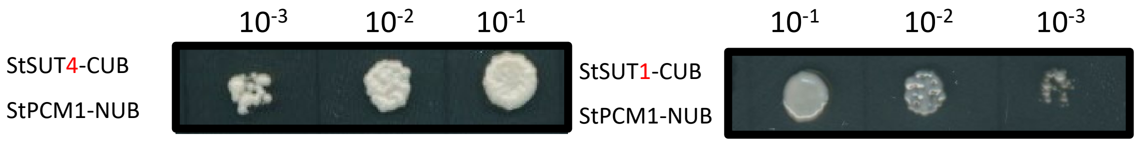

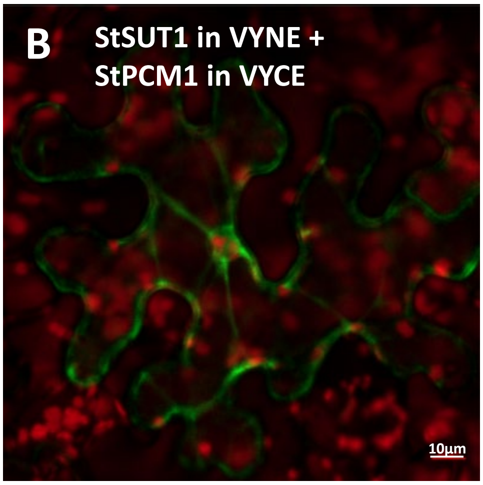

Supplemental Fig. S3. StPCM1 interactions. Not only StSUT4, but also StSUT1 is able to interact with StPCM1 in the split ubiquitin system **(A)** and in BiFC experiments **(B)**. The interaction of StSUT4 with the full-length cDNA of StPCM1 in split ubiquitin system and in BiFC experiments was used as a positive control (see Fig. 2). Interaction between the sucrose transporters and the calcium-binding protein PCM1 seems to take place in the cell periphery. Interaction strength between StSUT1 and StPCP1 is weaker than between StSUT4 and StPCM1.

Supplemental Fig. S4A

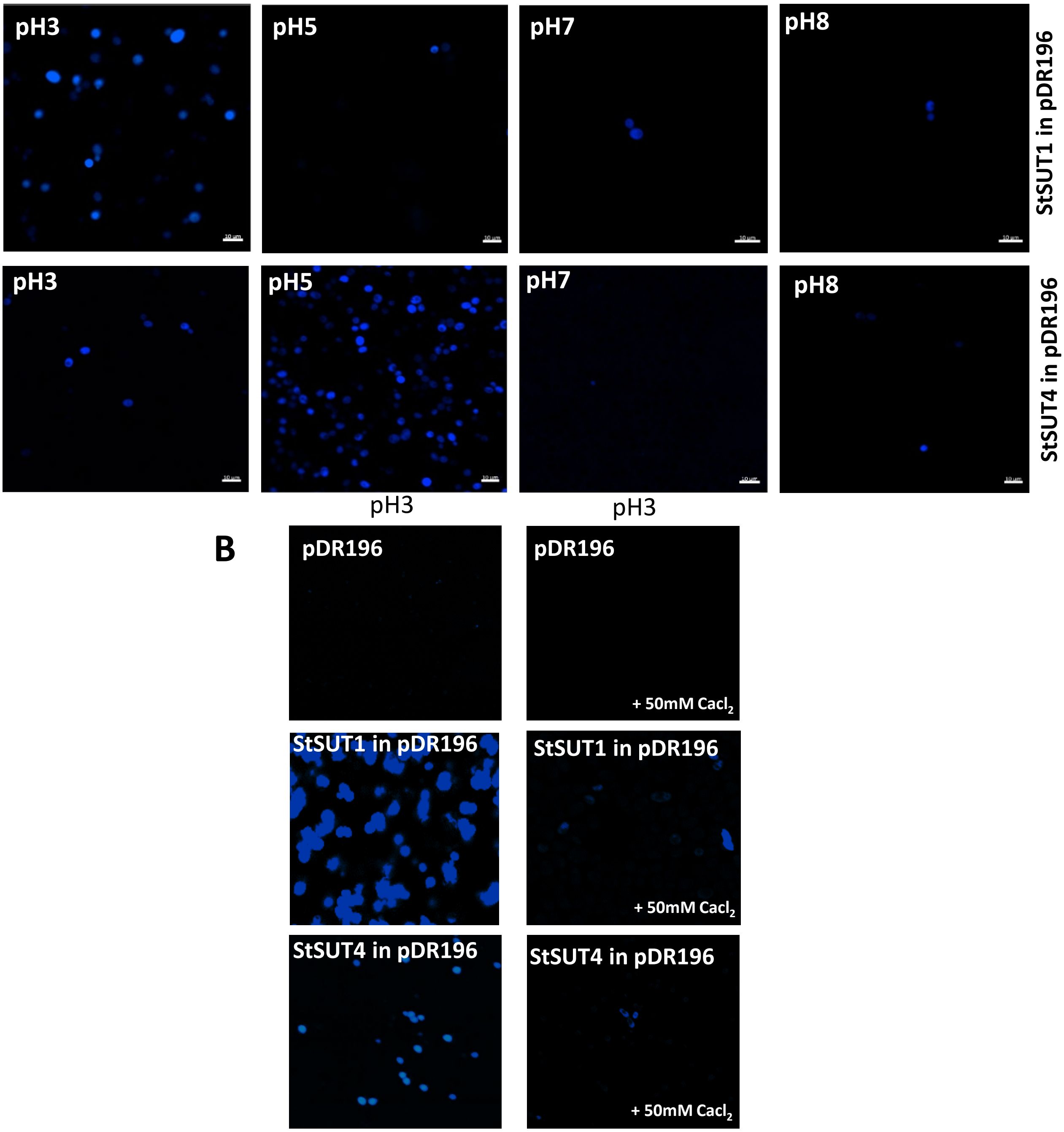

Supplemental Fig. S4. Microscopic analysis of yeast cells after esculin incorporation. Confocal images of yeast cells after esculin uptake assays. Esculin fluorescence was excited with 405 nm and emission was recorded in the blue range. **A.** Esculin uptake in yeast cells at various pH conditions revealed different pH optima for StSUT1 (at pH3) and for StSUT4 (at pH5) confirming quantitative esculin uptake measurements shown in Fig. 4. **B.** Esculin uptake by StSUT1 and StSUT4 in the absence (left panel) or presence (right panel) of 50 mM $CaCl_2$. Incubation with $CaCl_2$ occured for 1 h before uptake experiments. Yeast cells were thoroughly washed in medium without esculin before confocal microscopy. Scale bars correspond to 10 µm.

Supplemental Fig. S5A

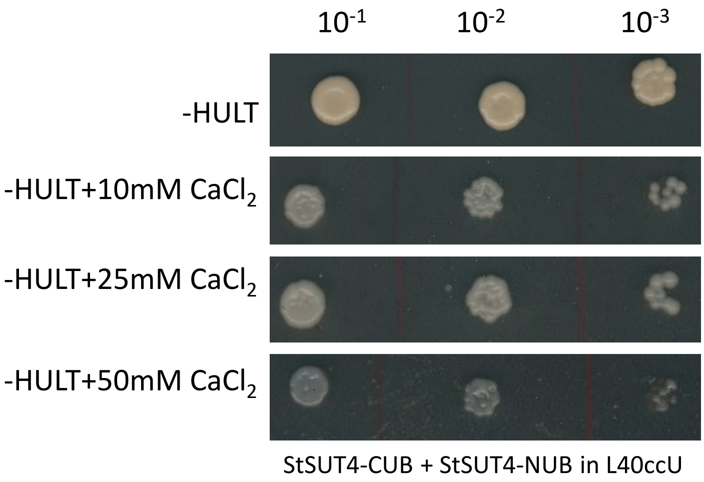

B

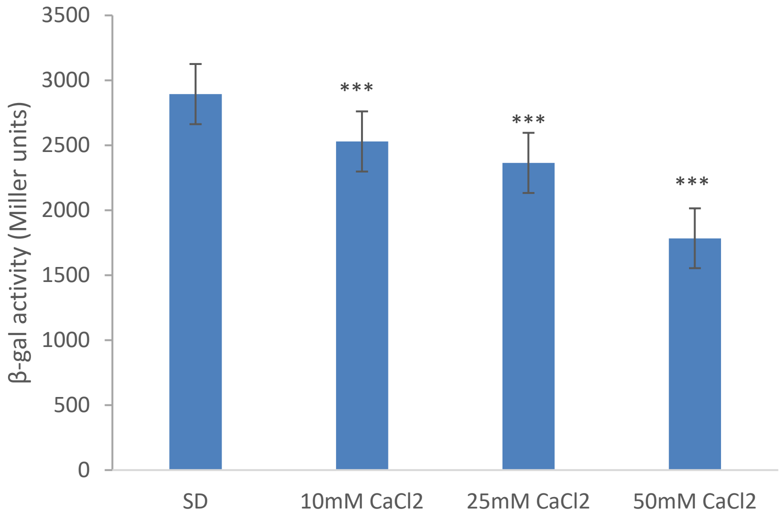

Supplemental Fig. S5. Homodimerization of StSUT4 in the presence of calcium. StSUT4 is able to form homo- and hetero-dimers (Reinders et al. 2000). Homodimerization of sucrose transporters is known to affect subcellular distribution (Garg et al. 2020). Here the effect of calcium ions on homodimer formation of StSUT4 is investigated by split ubiquitin system (**A**) as well as quantification of the interaction strength via quantitative evaluation of the β-galactosidase activity (**B**) in the presence of rising $CaCl_2$ concentrations. Increasing calcium concentrations are accompanied by decreasing homodimer formation and reduction of interaction strength. SD: minimal medium without $CaCl_2$.

Supplemental Fig. S6A

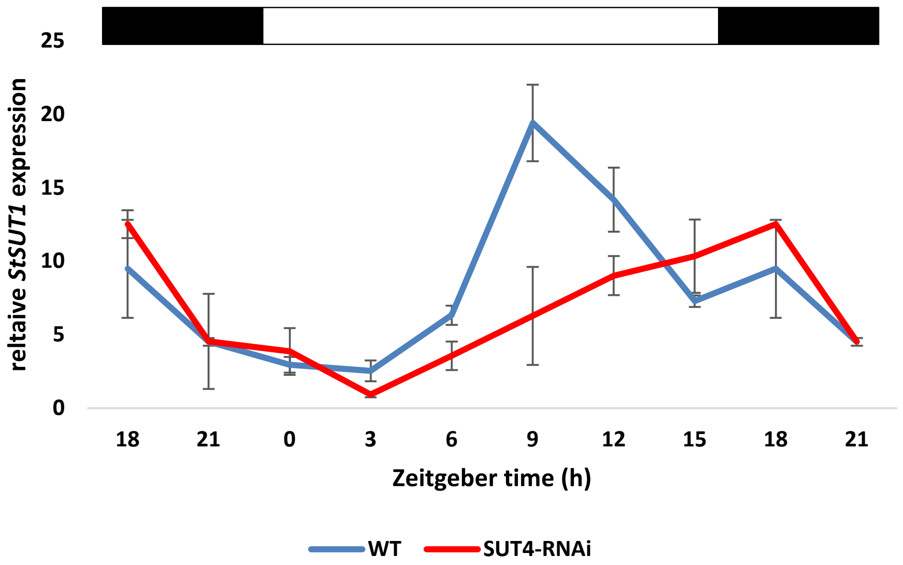

B

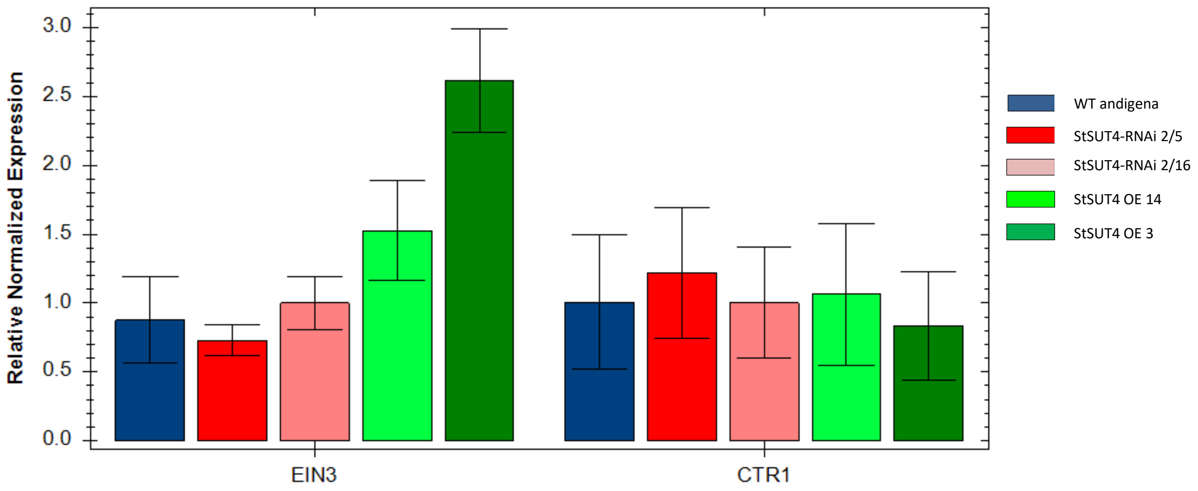

Supplemental Fig. S6 Expression analysis of *StSUT4*-RNAi plants A. Not only accumulation of soluble sugars is phase-delayed in *StSUT4*-RNAi plants (Chincinska et al. 2008), but also the expression of the main phloem loading sucrose transporter, *StSUT1*, shows phase-delayed oscillation and a decrease in amplitude under LD conditions as shown here by qPCR.
B. Main components of the ethylene signal transduction pathways have been analyzed in potato WT (blue bars) and *StSUT4*-RNAi (red bars) as well as StSUT4-GFP overexpressing plants (green bars). Whereas the transcription CTR1 does not show altered expression, the transcription inhibitor EIN3 is up-regulated when *StSUT4* is overexpressed suggesting a positive feedback loop via StSUT4.

Supplemental Fig. S7

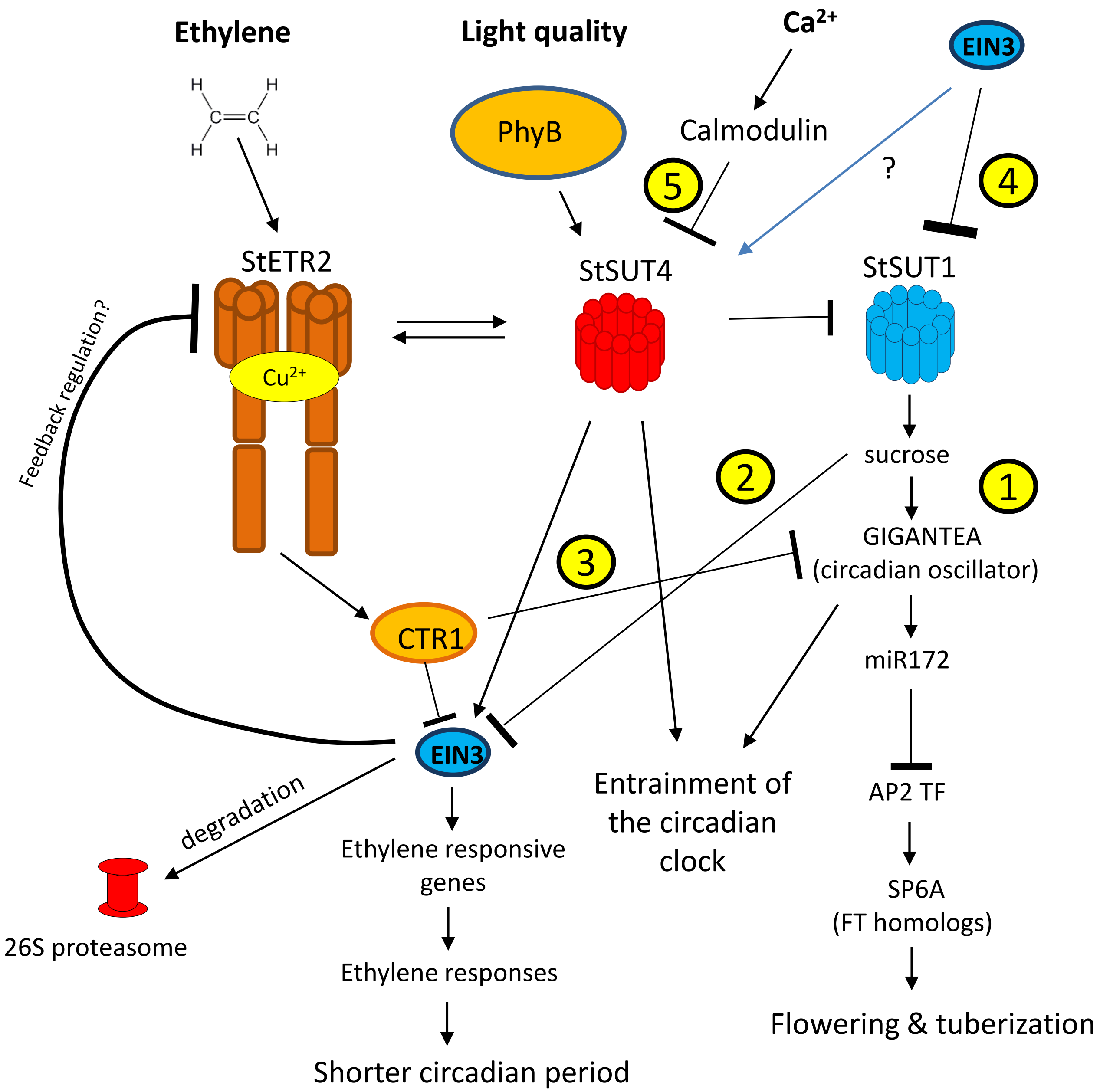

Supplemental Fig. S7. Hypothetical model illustrating the link between sucrose and ethylene signaling via StSUT4 and StETR2 interaction affecting the entrainment of the circadian clock. Sucrose accumulation has a positive impact on GI protein stability on the one hand (1), and negative impact via inhibition of EIN3 (2) which in the presence of ethylene induces ethylene responsive genes in the nucleus, or in the absence of ethylene is degraded via the 26S proteasome pathway. CTR1, the inhibitor of ethylene signaling, negatively affects GI protein stability (3) (with modifications according to Haydon et al. 2013).

StEIN3 expression is up-regulated in *StSUT4*-overexpressors (Fig. S6B). SUT1, SUT4 and ETR2 are EIN3 target genes in Arabidopsis and SUT1 expression is inhibited by direct EIN3 binding to the SUT1 promoter (4)(Chang et al. 2013). Here, a negative regulation of StSUT4 activity via calcium signaling is suggested (5).

Supplemental Fig. S8

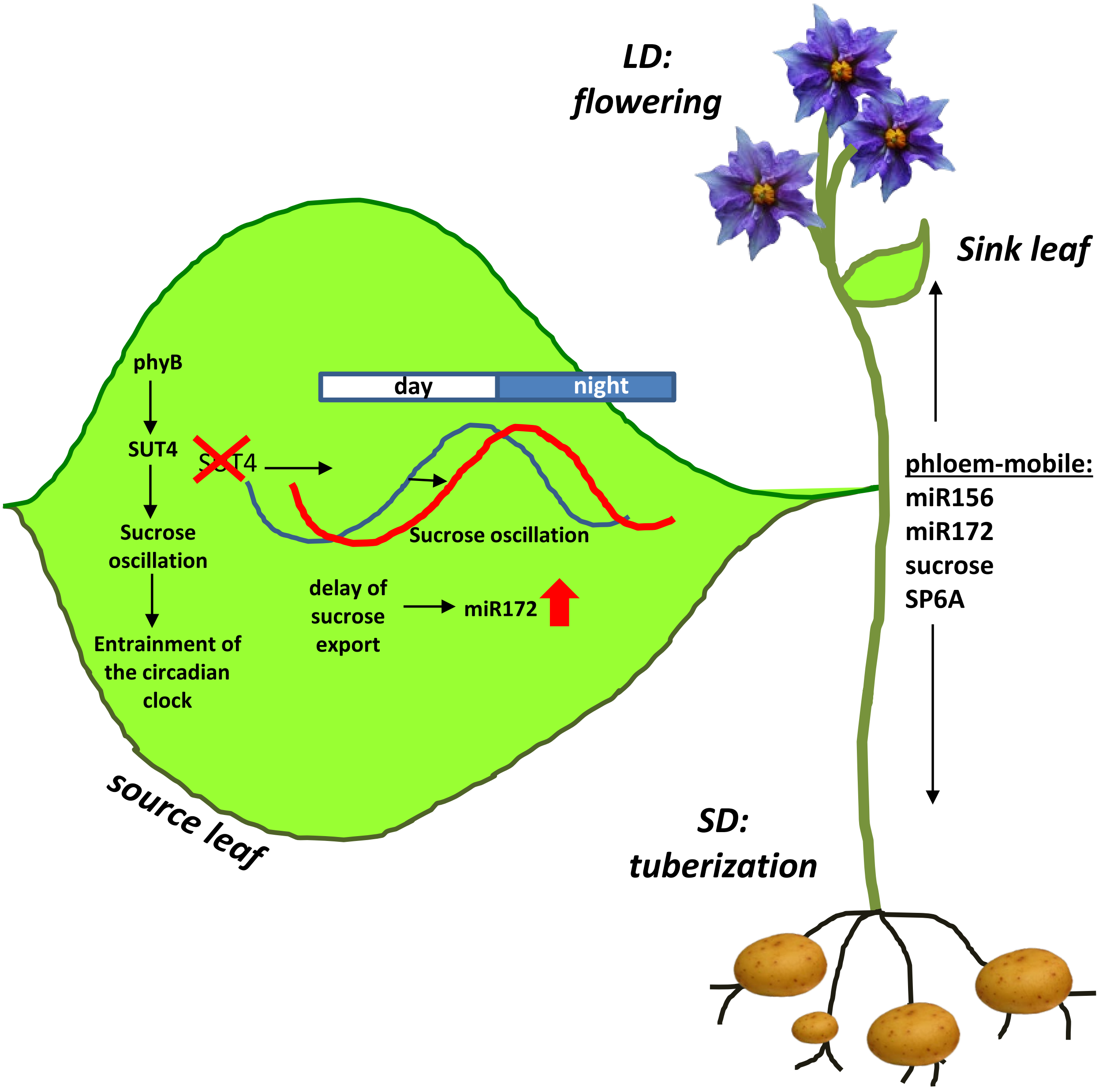

Supplemental Fig. S8. Hypothetical model illustrating the impact of sucrose in the entrainment of the circadian clock. Sucrose is required to adjust the phase of the circadian clock (Haydon et al. 2013). Inhibition of *StSUT4* expression causes a delay in sugar accumulation and sucrose export from source leaves of potato (Chincinska et al. 2008). miR172 levels are also changing during the day in potato (Garg et al. 2021). It is the question whether deregulation of *StSUT4* causes a shift in sucrose oscillation in leaves leading to out of phase-oscillation of sucrose responsive genes. The model is based on own experimental work (Chincinska et al. 2008, Garg et al. 2021) and knowledge about the importance of sucrose oscillation for the entrainment of the circadian clock observed in Arabidopsis (Haydon et al. 2013).

Supplementary Table 1: Primers used in this study.

Primers for GATEWAY cloning:

StSUT4 attB2 rev	AGAAAGCTGGGTTTCATGCAAAGATCTTGGGTTTC
StSUT4 attB1 fw:	AAAAAGCAGGCTTAATGCCGGAGATAGAAAGGCATAG
attB1:	GGGGACAAGTTTGTACAAAAAAGCAGGCT
attB2:	GGGGACCATTTGTACAAGAAAGCTGGGT

Sequencing primers:

ADH Prom:	CAGATGTCGTTGTTCCAGAGCT
upstream Met25YCgate:	CGTGTAATACAGGGTCGTCAGATAC
downstream Met25YCgate:	TCTGGAGG GATACCTGATCCAC

Primers used for insertion of the *ccdB* gene:

ccdB rev:	GAGACTGCAGCCCCTGGCCAGTGCACG
ccdB fw:	GAGACTGCAGATCTGGATTTTCAGCCTC

Primers for GATEWAY cloning of BiFC constructs:

StSUT1 fw:	AAAAAGCAGGCTTAAAAATGGAGAATGGTACAAAAAG
StSUT1 rev:	AGAAAGCTGGGTAATGGAAACCGCCCATGGCGAC
StSUT4 fw:	AAAAAGCAGGCTTAATGCCGGAGATAGAAAGGCATAG
StSUT4 rev:	AGAAAGCTGGGTTTGCAAAGATCTTGGGTTTCTC
StETR2 att fw:	AA AAAGCAGGCTTCATGGATTGTAACTGCTTTGATCCG
StETR2 att rev:	AGAAAGCTGGGTAAAGAACAGCCCTGTGCTCTAAAAGC

StPCM1 att fw:	AA AAAGCAGGCAAAATGGCAGAGCAGCTGACGG
StPCM1 att rev:	AGAAAGCTGGGTTCTTGGCAAGCATCATACGG

Primers used for cloning of prey cDNAs:

PCM1 fw:	GAGA GAATTC **atg** gca gag c ag ctg acg g
PCM1 rev:	GAGA GGATCC tca ctt ggc aag cat acg g
ETR2 fw:	GAGA GAATTC a**tg** gat tgt aac tgc ttt gat ccg
ETR2 rev:	GAGA GGATCC tta aag aac agc cct gtg ctc

Real time primers:

Ubi fw:	CACCAAGCCAAAGAAGATCA
Ubi rev:	TCAGCATTAGGGCACTCCTT
StETR2 fw:	GGCATGGTCCATGAATTGGAG
StETR2 rev:	AAATCATTACGCGCACGAAC
StPCM1 fw:	TGATGGCACGTAAGATGAAGG
StPCM1 rev:	TGCCTCTCGGATCATCTCCAT
